# Connecting Clinical Capacity and Intervention Sustainability in Resource-Variable Pediatric Oncology Centers in Latin America

**DOI:** 10.1007/s43477-023-00106-2

**Published:** 2023-11-14

**Authors:** Virginia McKay, Yichen Chen, Kim Prewitt, Sara Malone, Maria Puerto-Torres, Carlos Acuña-Aguirre, Yvania Alfonso-Carreras, Shilel Y. Alvarez-Arellano, Leticia A. Andrade-Sarmiento, Daniela Arce-Cabrera, Deiby Argüello-Vargas, Mariuxy D. C. Barragán-García, Rosario Batista-Del-Cid, Erika E. Blasco-Arriaga, Maria D. C. Cach-Castaneda, Gloria I. Ceballo-Batista, Mayra Chávez-Rios, Maria E. Costa, Maria E. Cuencio-Rodriguez, Rosdali Diaz-Coronado, Ever A. Fing-Soto, Teresa D. J. García-Sarmiento, Wendy C. Gómez-García, Cinthia J. Hernández-González, Yajaira V. Jimenez-Antolinez, Maria S. Juarez-Tobias, Esmeralda M. León-López, Norma A. Lopez-Facundo, Ruth A. Martínez Soria, Scheybi T. Miralda-Méndez, Erika Montalvo, Carlos M. Pérez-Alvarado, Clara K. Perez-Fermin, Monica L. Quijano-Lievano, Beatriz Salas-Mendoza, Edwin E. Sanchez-Fuentes, Marcia X. Serrano-Landivar, Veronica Soto-Chavez, Isidoro Tejocote-Romero, Sergio Valle, Elizabeth A. Vasquez-Roman, Juliana Texeira Costa, Adolfo Cardenas-Aguirre, Meenakshi Devidas, Douglas A. Luke, Asya Agulnik

**Affiliations:** 1. Brown School, Washington University, MSC 1196-251-46, 1 Brookings Drive, St. Louis, MO 63130, USA; 2. Department of Global Pediatric Medicine, St. Jude Children’s Research Hospital, Memphis, TN, USA; 3. Division of Population Health Sciences, Department of Surgery, Washington University School of Medicine, St. Louis, MO, USA; 4. Hospital Calvo Mackenna, Santiago, Chile; 5. Hospital Saint Damien, Port Au Prince, Haiti; 6. Benemérito Hospital General con Especialidades “Juan María de Salvatierra”, La Paz, Mexico; 7. Centro Medico Nacional Siglo XXI, Mexico, Mexico; 8. Hospital Pediatrico de Sinaloa, Culiacán, Mexico; 9. Hospital Nacional de Niños, San Jose, Costa Rica; 10. SOLCA Cuenca, Cuenca, Ecuador; 11. Hospital Jose Domingo De Obaldia, Barú, Chiriqui, Panama; 12. SOLCA Guayaquil, Guayaquil, Ecuador; 13. Hospital General Agustin O’Horan, Mérida, Mexico; 14. Hospital del Niño “Jose Renan Esquivel”, Panama, Panama; 15. Hospital para el Niño Poblano, Puebla, Mexico; 16. Hospital del Nino de la Santísima Trinidad, Cordoba, Argentina; 17. Hospital Universitario Austral, Buenos Aires, Argentina; 18. Instituto Nacional de Enfermedades Neoplasicas (INEN), Lima, Peru; 19. Hospital General Celaya, Celaya, Mexico; 20. Hospital de Especialidades Pediatricas, Tuxtla, Mexico; 21. Hospital Infantil Dr. Robert Reid Cabral, Santo Domingo, Dominican Republic; 22. Hospital Infantil Teleton de Oncologia (HITO), Querétaro, Mexico; 23. Hospital Universitario Dr. José E. González, Monterrey, Mexico; 24. Hospital Central Dr. Ignacio Morones Prieto, San Luis Potosí, Mexico; 25. Hospital Guillermo Almenara Irigoyen, Lima, Peru; 26. ISSEMYM Materno Infantil, Toluca, Mexico; 27. Hospital General de Tijuana, Tijuana, Mexico; 28. Hospital Escuela Universitario, Tegucigalpa, Honduras; 29. SOLCA Quito, Quito, Ecuador; 30. Centro Estatal de Cancerologia, Xalapa, Mexico; 31. Hospital Infantil Regional Universitario Dr. Arturo Grullon, Santiago, Dominican Republic; 32. Centro Médico Imbanaco, Cali, Colombia; 33. Hospital del Niño Manuel Ascencio Villarroel, Cochabamba, Bolivia; 34. Hospital Nacional de Ninos Benjamin Bloom, San Salvador, El Salvador; 35. Hospital Caja Petrolera de Salud Santa Cruz, Santa Cruz, Bolivia; 36. Hospital Civil de Guadalajara, Guadalajara, Mexico; 37. Hospital para el Nino (IMIEM), Toluca, Mexico; 38. Unidad Nacional de Oncologia Pediatrica (UNOP), Guatemala, Guatemala; 39. Hospital Nacional Edgardo Rebagliati Martins, Lima, Peru

## Abstract

Clinical capacity for sustainability, or the clinical resources needed to sustain an evidence-based practice, represent proximal determinants that contribute to intervention sustainment. We examine the relationship between clinical capacity for sustainability and sustainment of PEWS, an evidence-based intervention to improve outcomes for pediatric oncology patients in resource-variable hospitals. We conducted a cross-sectional survey among Latin American pediatric oncology centers participating in Proyecto Escala de Valoración de Alerta Temprana (EVAT), an improvement collaborative to implement Pediatric Early Warning Systems (PEWS). Hospitals were eligible if they had completed PEWS implementation. Clinicians were eligible to participate if they were involved in PEWS implementation or used PEWS in clinical work. The Spanish language survey consisted of 56 close and open-ended questions about the respondent, hospital, participants’ assessment of clinical capacity to sustain PEWS using the clinical sustainability assessment tool (CSAT), and perceptions about PEWS and its use as an intervention. Results were analyzed using a multi-level modeling approach to examine the relationship between individual, hospital, intervention, and clinical capacity determinants to PEWS sustainment. A total of 797 responses from 37 centers in 13 countries were included in the analysis. Eighty-seven percent of participants reported PEWS sustainment. After controlling for individual, hospital, and intervention factors, clinical capacity was significantly associated with PEWS sustainment (OR 3.27, *p* < .01). Marginal effects from the final model indicate that an increasing capacity score has a positive influence (11% for every additional CSAT point) of predicting PEWS sustainment. PEWS is a sustainable intervention and clinical capacity to sustain PEWS contributes meaningfully to PEWS sustainment.

## Introduction

Failure to sustain effective clinical interventions results in poor outcomes, including waste of initial investments and loss of patient benefits, which is particularly problematic in low-resource settings where resources for intervention implementation are limited (Hodge & Turner, 2016; Iwelunmor et al., 2016; Rabin & Brownson, 2017). Sustainment, or the continued use of an intervention over time with associated positive health outcomes, is critical to maximizing the long-term benefits of evidence-based interventions (Moore et al., 2017; Scheirer & Dearing, 2011; Shelton & Lee, 2019; Shelton et al., 2018). However, little scientific evidence identifies sustainability drivers or determinants, particularly in low-resource settings (Iwelunmor et al., 2016).

Theoretically, organizational capacity for sustainability is a proximal collection of determinants that promotes intervention sustainment (Schell et al., 2013). Organizational capacity describes the resources needed to implement and sustain an intervention, such as staff, financial support, or leadership support. While the capacity to provide an evidence-based intervention is conceptually similar across settings, certain factors are more relevant to promote sustainability specifically in clinical settings (Malone et al., 2021). To date, studies examining determinants of clinical capacity for sustainability include primarily high-resource hospitals and are largely theoretical (Birken et al., 2020; Cowie et al., 2020; Hailemariam et al., 2019).

An additional important challenge in empirically examining sustainability determinants is the need of valid and reliable measures. A recent sustainability review noted that most measures are only used once and identified few measures used in multiple countries or available in multiple languages (Hall et al., 2022). Through our previous work, we have established a measure, the Clinical Sustainability Assessment Tool (CSAT) based on seven theoretical domains describing clinical capacity for sustainability consisting of engaged staff and leadership, engaged stakeholders, organizational readiness, workflow integration, implementation and training processes, and outcomes and effectiveness. The CSAT has demonstrated good reliability and validity in a mix of clinical contexts. Furthermore, users have rated the measure as easy to use and freely available (Malone et al., 2021).

In this study, we continue to build on the validity and utility of the CSAT as a measure of clinical capacity for sustainability by examining clinical capacity to sustain Pediatric Early Warning System (PEWS). PEWS are evidence-based interventions that aid in the early identification of clinical deterioration in children with cancer receiving treatment and improve patient outcomes in low-resource hospitals (Agulnik et al., 2017a, 2017b, 2019, 2023; Brown et al., 2019; Garza et al., 2021; Graetz et al., 2020, 2021). While survival for childhood cancer in high-resource settings is more than 80%, over 90% of children with cancer live in low-resource settings, where hospitals have limited material and human resources to provide acute medical care (Arias et al., n.d.; Muttalib et al., 2021). Subsequently, children with cancer in low-resource settings have high mortality, particularly if they experience clinical deterioration during cancer treatment (Agulnik et al., 2021a, b, c; Wösten-van Asperen et al., 2019). Implementation of PEWS in these settings has shown to have multi-level benefits for patients, providers, medical teams, and institutions (Mirochnick et al., 2022). These benefits include a reduction in clinical deterioration events and intensive care unit (ICU) utilization (Agulnik et al., 2017a, 2017b), improved interdisciplinary and family communication, provider empowerment and perceived quality of care (Garza et al., 2021; Graetz et al., 2020, 2021), and result in an annual cost savings of over US$350,000 per hospital (Agulnik et al., 2019).

While these and other studies demonstrate the effectiveness of PEWS to improve childhood cancer treatment outcomes, this evidence-based intervention is underutilized in low-resource settings, partly due to implementation challenges (Agulnik et al., 2022a, b, c; Nyandat et al., 2022). To address these barriers, St. Jude Children’s Research Hospital (St. Jude) partnered with regional stakeholders in Latin America to develop Proyecto EVAT, a quality improvement collaborative to support implementation of PEWS (Agulnik et al., 2022a, b, c). This initiative has successfully promoted scale-up of PEWS among childhood cancer centers in the region, with over 40 centers successfully implementing the intervention since 2017 (Agulnik et al., 2022a, b, c).

The impact of PEWS, however, depends not only on successful implementation; but sustained PEWS use over time for long-term benefits. Earlier work by our team suggests centers face a range of challenges sustaining PEWS, including waning leadership interest, staff turnover, inadequate material resources for PEWS use, and external health systems disruptions, such as the COVID-19 pandemic (Agulnik et al., 2022a, b, c). Given these challenges, it is necessary to establish a better understanding of clinical capacity factors most related to sustaining PEWS over time.

In this study, we evaluate the relationship between hospital, clinician, and PEWS characteristics, clinical capacity, and PEWS sustainment in Proyecto EVAT centers that successfully implemented and are ideally sustaining PEWS. The goal of the current study is twofold: (1) to examine the relationship between clinical capacity and PEWS sustainment after controlling for other common sustainability determinants and (2) to determine whether some domains of clinical capacity are more relevant for sustaining PEWS than others.

## Methods

We conducted a cross-sectional survey with clinicians using PEWS in pediatric oncology centers in Latin America participating in the Proyecto EVAT improvement collaborative. Data were collected in the spring of 2022 through an anonymous electronic survey that asked questions about the participants’ characteristics, perceptions of PEWS and PEWS use as an intervention, and clinical capacity to sustain PEWS at their center. The Institutional Review Board approved all study protocols at St. Jude Children’s Research Hospital (St. Jude) as exempt human subjects’ research.

### Intervention

Escala de Valoración de Alerta Temprana (EVAT) is a validated Spanish-language PEWS adapted for low-resource settings (Agulnik et al., 2016, 2017a, 2017b). This PEWS includes a 5-component scoring tool (neurologic, cardiovascular, respiratory, staff, and family concern) based on a patient’s vital signs, physical examination findings, and treatment requirements (Agulnik et al., 2017a, 2017b). Using this tool, hospitalized patients are scored 0 to 11 by a bedside nurse during routine vital sign assessments. Higher scores indicate potential clinical deterioration and are addressed following an action algorithm that guides the clinical team in appropriate escalation of care.

### Implementing Hospitals

EVAT adoption and implementation are supported through Proyecto EVAT, a multicenter quality improvement collaborative in Latin America to improve outcomes for children with cancer who experience critical illness through the implementation of PEWS (St. Jude Global, n.d.). Hospitals providing childhood cancer care in Latin America learn about Proyecto EVAT from collaborating with St. Jude Global or through other participants and apply to join an annual cohort. Participating centers receive mentorship through a standardized implementation process to adopt PEWS, including initial training, implementation planning, and piloting, and initial implementation. At each participating hospital, implementation completion, meaning the hospital has successfully adopted and implemented PEWS, is defined as having at least two months of high-quality PEWS implementation with fidelity, defined as having less than 15% errors in 3 types of PEWS use, specifically omissions (not documenting PEWS with routine vital signs), calculation errors, and algorithm non-adherence, as measured through weekly documentation quality checks by local teams (Agulnik, Gonzalez Ruiz, et al., 2022b). Following this milestone, centers independently sustain PEWS use without support from Proyecto EVAT, but continue to collaborate with the initiative in research studies or by mentoring new centers. Currently, Proyecto EVAT encompasses 80 diverse pediatric oncology centers in 20 countries, with 10 new centers joining annually; at the time of this study (March, 2022), 43 centers had completed PEWS implementation (Agulnik, Garza, et al., 2021).

Proyecto EVAT is managed by St. Jude with oversight by a Steering Committee composed of 28 multidisciplinary experts in PEWS implementation from the region. The current project was presented to the Proyecto EVAT Steering Committee for approval before beginning data collection. Members were allowed to review data collection protocols and measures and provide feedback to the research team for incorporation.

### Eligibility and Recruitment

Forty-three hospitals completed the PEWS implementation as of March 2022 and were initially eligible. Three centers who had completed a capacity assessment in the prior 6 months were excluded to reduce survey burden on participants for a total of 39 eligible hospitals. This study was described to local PEWS implementation team leaders who were asked to generate an email list of eligible clinicians involved in implementing or using PEWS at their center. Potential participants were then contacted by email by a research team member with a link to an anonymous electronic survey. Participants had three to four weeks to complete the survey and were sent weekly reminders. Participants received no individual incentives; however, each participating center received a CSAT report summarizing responses at their center (Agulnik, Malone, et al., 2021).

### Data Collection and Management

Data were collected using the electronic survey platform Qualtrics. Respondents were asked 56 close- and open-ended questions. The survey took approximately 15–20 min to complete, and data were collected anonymously to protect respondent identity and reduce desirability bias. Data were then merged with hospital characteristics reported by implementation site leads.

#### Measures

This study used a Spanish-language survey previously piloted with Proyecto EVAT centers (Agulnik, Malone, et al., 2021). Survey questions fell into four broad categories: questions about the respondent (demographics, seven items), the hospital (organization, six items), the participants assessment of clinical capacity to sustain PEWS (CSAT, 35 items), and perceptions about PEWS and its use with pediatric oncology patients at their center (intervention, eight items). Items and response options asked of the respondent and hospitals can be found in Table 1 and perceptions about PEWS can be found in Table 2.

Clinical capacity for sustainability was assessed across seven CSAT domains with five questions per domain scored on a Likert scale of 1 (low capacity) to 5 (high capacity) (Malone et al., 2021). This measure is based on the Clinical Capacity for Sustainability Model, which outlines seven clinical capacities for sustainability domains: (1) engaged staff and leadership—frontline and administrative staff who are supportive of the intervention; (2) engaged stakeholders—other individuals, such as patients or parents, who are supportive of the intervention; (3) organizational readiness—organizational internal support and the resources needed to manage the intervention effectively; (4) workflow integration—how well the intervention fits into work that is done or will be done; (5) implementation and training—the process of implementing and training to deliver and maintain an intervention; (6) monitoring and evaluation—a process to evaluate the intervention to determine its effectiveness; and (7) outcomes and effectiveness—using monitoring and evaluation to determine outcomes for clinicians or patients.

The CSAT measure was previously translated to Spanish from the original measure using a rigorous process and piloted with Proyecto EVAT centers, demonstrating high reliability (Agulnik, Malone, et al., 2021). The dependent variable indicating PEWS sustainment was the frequency of PEWS use (“Regarding patients under my care, how often is PEWS used in their care?”), with “all of the time” indicating high sustainment and all other responses indicating low sustainment.

### Data Analysis

Descriptive statistics were used to summarize individual, hospital, and intervention-level characteristics. Overall and domain-specific CSAT scores were calculated as an average for each participant. The primary outcome measure of PEWS sustainment was defined by dichotomizing the frequency of PEWS use, to all the time (sustained) and less than all the time (not sustained). The utilization of PEWS with every vital sign check with every hospitalized pediatric oncology patient post-implementation is the expectation, and there were very few respondents who reported using PEWS less than all of the time making the dichotomization appropriate for the anticipated analyses. For analytic purposes, individual characteristics (profession, main area of work, PEWS implementation role, and length of work at hospital), hospital characteristics (hospital type), and intervention-level characteristics (strength of evidence supporting PEWS, importance of PEWS to providing quality care, and difficulty of PEWS implementation) were each collapsed into fewer categories to have sufficient power for statistical modeling.

**Figure 1:**
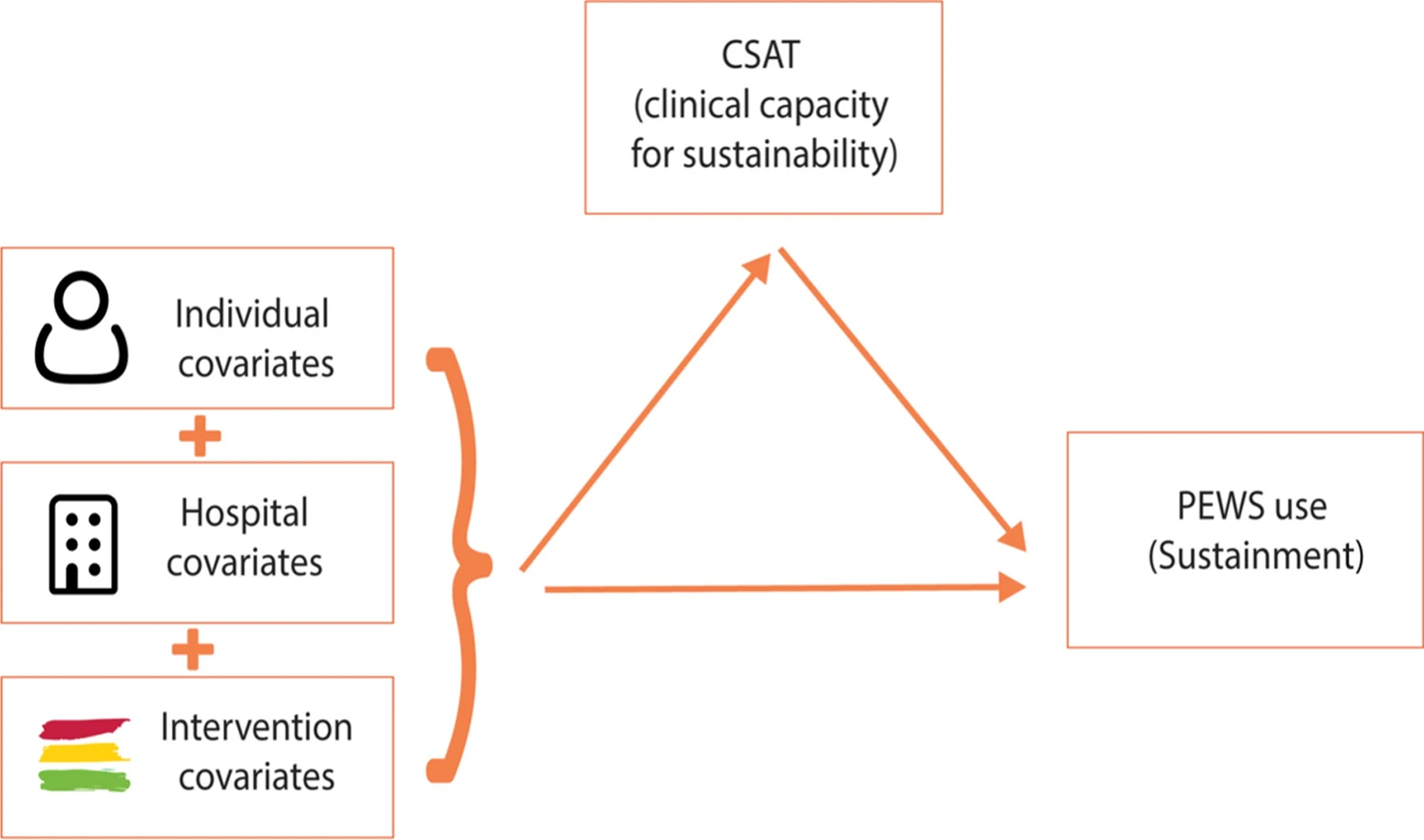
Conceptual model of variables

A Wilcoxon rank sum test was used to compare clinical capacity scores between the two categories of the primary outcome. Mixed-effects multi-level modeling was conducted iteratively, by adding blocks of individual-, organization-, and intervention-level variables. The final model added the primary explanatory variable of interest, clinical sustainability capacity. Figure 1 illustrates how the final model and variables are conceptualized. Secondary mixed-effects models were also examined with the separate CSAT subdomains. Model performance was evaluated by Akaike information criterion (AIC). F test was used to compare two nested models. Marginal effects were calculated for the significant variables in the final model to explore their influence on PEWS sustainment further. Data were managed and analyzed using R, version 4.2.2 and SAS, version 9.4.

## Results

**Figure 2:**
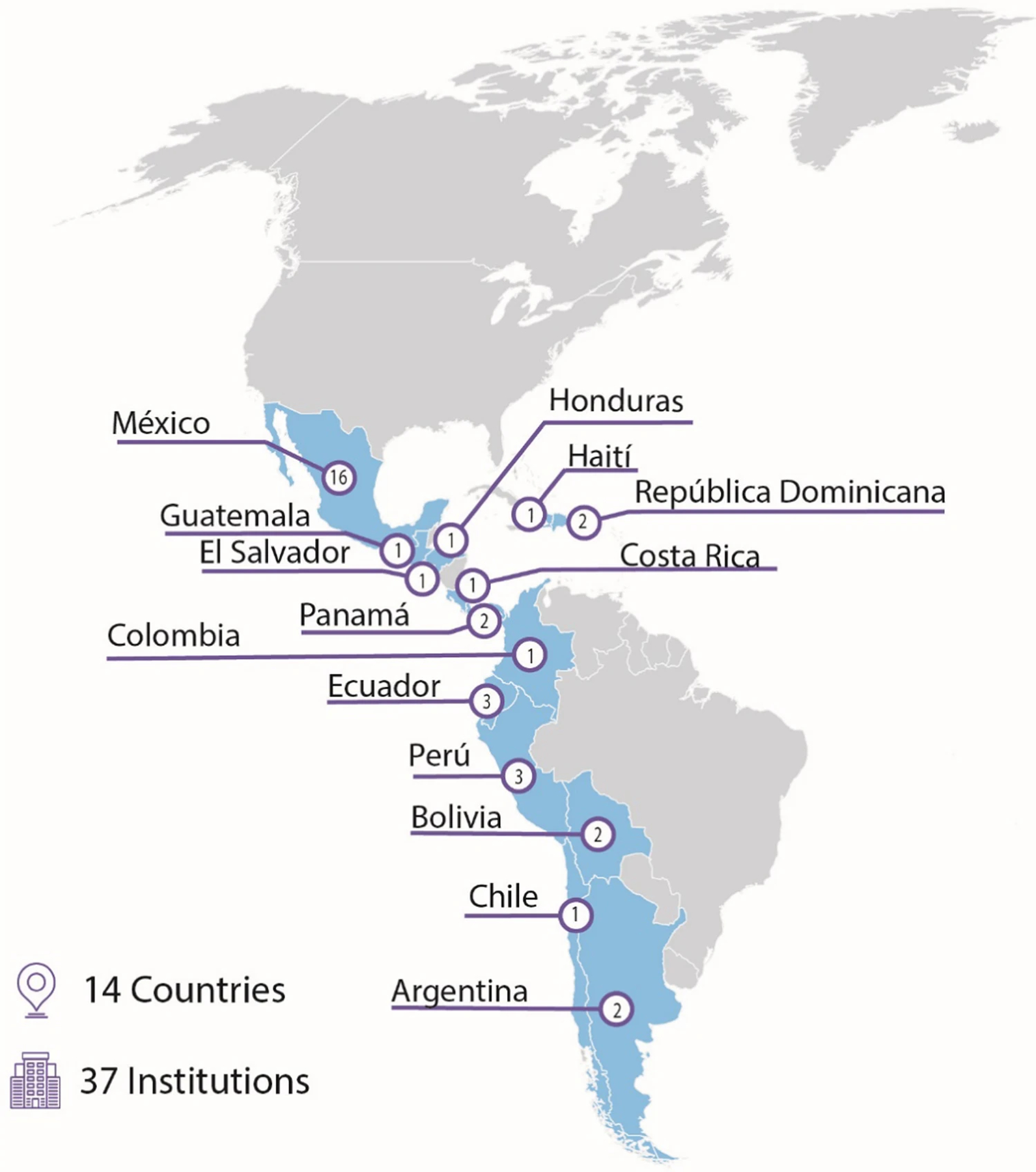
Participating centers by country

Of 39 Proyecto EVAT hospitals completing PEWS implementation as of March 2022, two centers declined to participate in this study. Of the remaining 37 centers in 13 countries in Latin America, 72.7% of eligible individuals responded per hospital (range 32% to 100%), achieving 800 individual responses (Fig. 2). Three responses were excluded due to marking “not applicable” on the primary outcome (PEWS sustainment), resulting in 797 responses used in analysis. Participant and hospital characteristics are provided in Table 1. Briefly, participants were majority female (82%), serving as a nurse (57%), serving on the hospital floor (87%), were using PEWS but not involved in implementing PEWS (72%), and had been working at their hospital for 5 years or less (40%). Hospitals were mostly located in an upper middle-income country (84%), identified as either a general or women and children’s hospital (43%) were publicly funded (73%), served as a teaching hospital (95%), had an average 1:6 nurse-to-patient ratio (range 3 to 10 patients), and provided care for 89 new pediatric oncology diagnoses a year (range 5 to 800). Finally, hospitals completed PEWS implementation on average 28 months (range 4–88 months) prior to this study.

Eighty-seven percent of participants reported PEWS being used all the time during patient care, indicating high PEWS sustainment. Participants were asked to evaluate PEWS as an intervention (Table 2). Most respondents rated the strength of the evidence supporting PEWS as strong or very strong (90%) and important or very important to providing quality care to patients (98%). Respondents reported PEWS implementation as mixed, with 35% reporting PEWS implementation as difficult, 24% as neither easy nor difficult, and 36% as easy. Individuals rated clinical capacity to sustain PEWS ranging from 1.1 to 5 (of 5 maximum, with 5 being the highest capacity) with hospitals averaging a 4.25 (range 3.7–4.7).

**Figure 3:**
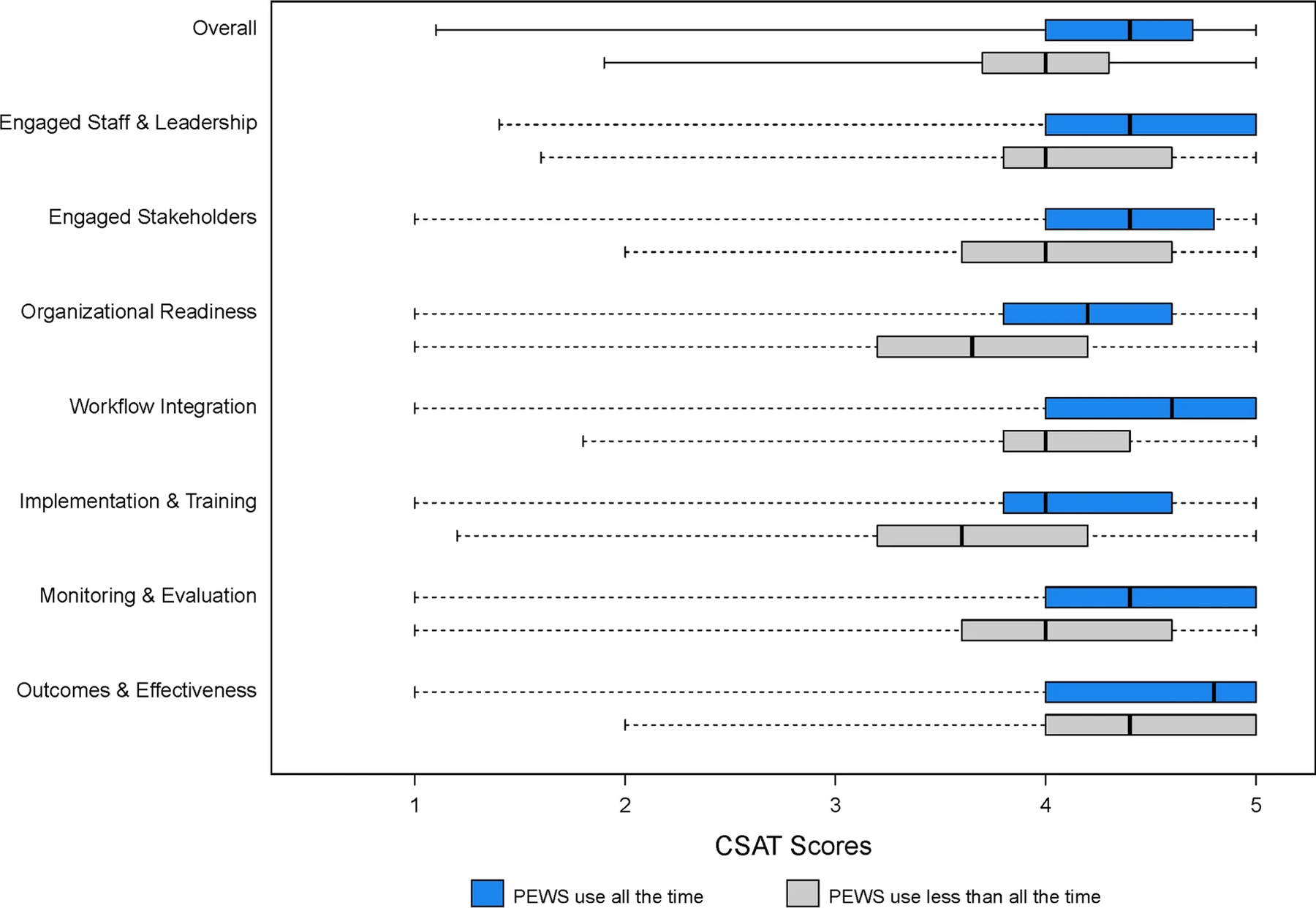
Box plots of overall and domain-specific CSAT scores. Note scores are separated by clinicians reporting PEWS sustainment (all of the time) in blue and lack of PEWS sustainment (PEWS use less than all the time) in gray

Figure 3 presents the capacity score for those who reported PEWS sustainment compared to those who did not. Most respondents rated clinical capacity across all domains between three and five with a similar overall capacity score. However, participants who reported sustaining PEWS reported significantly greater clinical capacity overall and across all domains (p < .01).

### Capacity and PEWS Sustainment

After assessing univariate and bivariate statistics, we employed multi-level mixed-effects modeling to examine the relationship of clinical capacity with PEWS sustainment. We began with a (1) null model (no covariates), (2) a model adding individual-level covariates, (3) a model adding hospital-level covariates, and (4) a model adding intervention covariates. The final two models were designed to evaluate the relationship between clinical capacity and PEWS sustainment as an overall score and by capacity domain. More specifically, these were (5) a multi-level model adding overall clinical capacity scores and (6) a multi-level model separating overall capacity into individual domain scores for clinical capacity. The final models are summarized in Table 3, including intercept coefficients, odds ratios (OR) for covariates, and significance values.

### Null, Individual, Hospital, and Intervention Models (Models 1–4)

A table summarizing the outcomes for all statistical models are included as Supplemental File B. Briefly, the interclass correlation was calculated from the null model (Model 1) was 0.23, indicating 23% variability among individual responses by hospital-level clustering. It also indicates appropriateness of multi-level modeling as an analytic approach. For the model incorporating individual characteristics (Model 2), all individual-level covariates were added as level one variables to the model. The AIC for the model was 573.07. Three covariates, role in the hospital (p < .01), role in PEWS implementation (p = .02), and length of time working at the hospital (p < .01) were significant. Doctors were less likely to report PEWS sustainment than nurses (OR .32, p < .01). PEWS implementation leaders were more likely to report PEWS sustainment compared to clinical staff (OR 2.64, p < .01). Staff working at the hospital no more than 5 years or from 6 to 10 years were more likely to report PEWS sustainment compared to staff working at hospitals more than 10 years (OR 2.82, p < .01and OR 2.96, p < .01, respectively). All other individual-level variables explored were non-significant (see Table 1 for a list of covariates).

For the model incorporating hospital characteristics (Model 3), all hospital-level covariates were added as level two variables to the model simultaneously. The AIC increased to 584.95. Result of F test (F .80, df1 = 4, df2 = 8, p = 0.56) indicates no significant difference between model 2 and 3. Covariates in the individual model (Model 2) remained significant, and additional significant covariates were annual diagnoses. Participants from hospitals with fewer annual diagnoses were more likely to report complete PEWS sustainment (OR 0.78, p = .03). All other hospital-level variables explored were non-significant (Table 1).

For the model incorporating intervention characteristics (Model 4), all intervention-level covariates (See Table 2 for a list of covariates) were added as level one variables to the model at the same time. AIC decreased slightly to 582.69. Result of F test (F 1.90, df1 = 3, df2 = 11, p = .19) indicates no significant different between Model 4 and 3. In addition to covariates significant in Model 3, the perceived importance of PEWS was significant. Participants who perceived PEWS as important were more likely to report PEWS sustainment than those did not (OR 11.14, p < .01).

### Overall Clinical Capacity (Model 5)

The results for Models 5 and 6 are summarized in Table 3. In summary for Model 5, individual reports of overall clinical capacity (CSAT) score were added as a level one variable to the model. AIC decreased to 554.53 indicating better model fit. Result of F test (F 6.90, df1 = 1, df2 = 12, p = .02) indicates a significantly better fit of model 5 compared to model 4. Participant role as a PEWS implementation leader at the individual level and the annual number of diagnoses at the hospital level was no longer significant in this model. Clinical role and time working at the hospital at the individual level and perceived PEWS importance remained significant. The higher overall clinical capacity score was significantly associated with individuals who reported PEWS sustainment (OR 3.27, p < .01). That is, an individual reporting higher clinical capacity was more likely to fully sustain PEWS.

**Figure 4:**
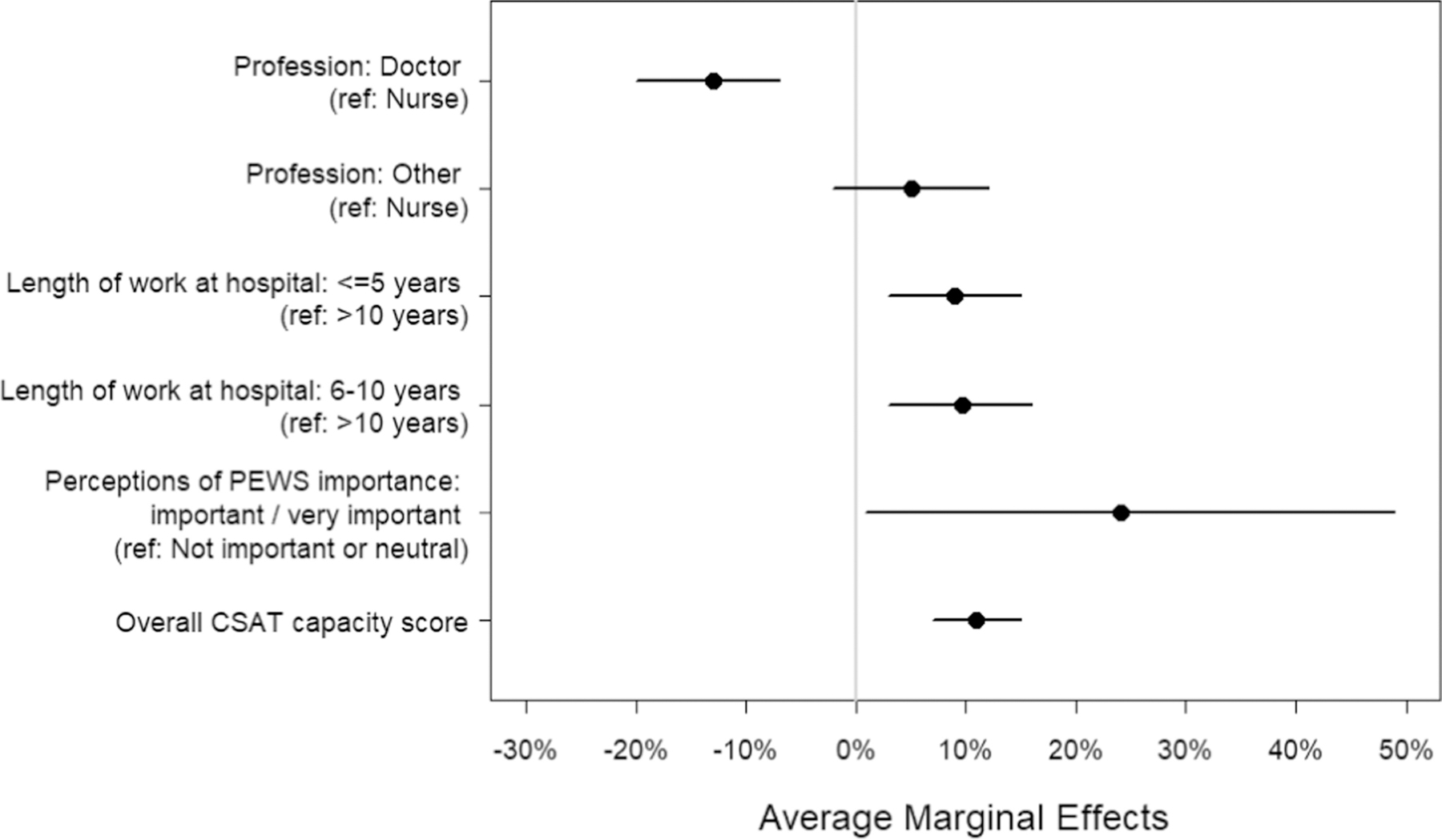
Marginal effects plot including significant variables from final mixed-effects model

We used a marginal effects plot to further examine the influence of significant variables in the overall clinical capacity model on PEWS sustainment (Fig. 4). In alignment with our multi-level model results, doctors had a 13% lower probability of reporting PEWS use all of the time compared to nurses and clinical staff who had been working at the hospital for no more than 5 years and for 6 to 10 years had a 9% higher probability of reporting sustained PEWS use compared to those who had been working at the hospital for longer than ten years. While perceptions of PEWS importance to providing quality clinical care had the highest probability improvement (24%) of predicting the likelihood of PEWS use all of the time among respondents, we also observed a larger variety of average marginal effects (13% standard error) for this variable. This is likely due to low variability in the responses for this question (i.e., 98% respondents rated PEWS as important or highly important; see Table 2), suggesting an abundance of caution when interpreting the influence of this variable on PEWS use. Finally, increasing clinical capacity score had a positive influence (11% per one unit increasing) of predicting PEWS sustainment.

### Individual Capacity Domains

We explored the association of individual reports of clinical capacity domains with PEWS sustainment in a sixth model (Model 6). Overall capacity scores were removed and replaced with individual clinical capacity domain scores again as a level one variable. The AIC decreased to 548.62 indicating a better model fit. Significance for individual- and intervention-level variables remained the same from Model 5. Among individual clinical capacity domains, organizational readiness and workflow integration were significantly related to PEWS sustainment. Individuals reporting higher score in organizational readiness or workflow integration were more likely to report PEWS sustainment (OR 2.02, *p* < .01 and OR 2.70, *p* < .01, respectively).

In summary, most respondents reported sustaining PEWS in the care of children with cancer. Capacity for sustainability was positively associated with PEWS sustainment in our final model. Furthermore, marginal effects plots demonstrated that for every point increase in capacity score, reported PEWS sustainment increased 11%. When exploring specific capacity domains, organizational readiness and workflow integration were significantly associated with PEWS sustainment. Several individual, hospital, and intervention-related variables were associated with PEWS sustainment in initial models (Models 1–4). However, few of these covariates remained significant after adding clinical capacity (CSAT score). Significant covariates in the multivariable model included clinician role, length of time working at the hospital, and perceptions of PEWS; hospital-level covariates were not significantly associated with PEWS sustainment.

## Discussion

Supporting intervention sustainability can significantly improve health and well-being for communities, especially those living in low-resource settings. Our work continues to contribute to the body of scientific literature identifying determinants of intervention sustainment and the contribution of clinical capacity for sustainability as a driver of successful intervention sustainment. It also lends additional validity to CSAT as a measure of clinical capacity across geographic and resource-diverse settings.

Our analysis suggests that PEWS was highly sustainable among individual respondents over 2 years after completing PEWS implementation and that many respondents felt hospitals had sufficient clinical capacity for PEWS sustainment despite largely identifying as low resourced. Doctors (compared to nurses) and clinical staff who had been working at hospitals for more than 5 years were less likely to report PEWS sustainment. One plausible hypothesis is that while doctors are important for clinical care and evaluation of hospitalized pediatric oncology patients with abnormal PEWS, PEWS calculation and documentation are performed typically by bedside nurses. As such, doctors may not be aware of how routinely PEWS is being used with patients in their hospitals. Similarly, participants with greater than 10 years working at their center may be in leadership and less patient care facing roles, potentially explaining their lower perception of continued PEWS use. Importantly, hospital-level characteristics, including hospital organization, funding, or country income level, were not significantly associated with PEWS sustainment. These results are an encouraging indication that clinical capacity is the primary driver of PEWS sustainment and helps demonstrate PEWS as a sustainable evidence-based intervention regardless of hospital resource level. This work provides further evidence to support the global scale-up of PEWS as a sustainable intervention to improve treatment for pediatric oncology patients worldwide.

Overall, our results demonstrated that clinical capacity for sustainability was significantly related to the sustainment of this intervention using an established measure, the CSAT. These results lend additional validity to the measure and its applicability across multiple clinical settings. While clinical capacity overall was related to intervention sustainment, our results suggest that specific domains had greater influence than others. Specifically, organizational readiness (organizational internal support and the resources needed to manage the intervention effectively) and workflow integration (how well the intervention fits into work that is done or will be done) were significantly related to PEWS sustainment. These findings fit with organizational factors needed to sustain a clinical, multidisciplinary intervention that must be integrated into routine care and are consistent with past work describing common challenges in PEWS sustainment (Agulnik, Schmidt-Grimminger, et al., 2022c). These findings suggest that PEWS sustainability is impacted by modifiable factors amendable to strategies that develop capacity for sustainability, rather than fixed institutional structural characteristics. In addition, these domains represent potential targets for future implementation strategies designed to improve PEWS sustainability.

Although organizational readiness and workflow integration were important for PEWS sustainment, we expect that these relationships may not hold for different types of interventions in different contexts as others have recognized (Scheirer, 2013). Our respondents rated PEWS as highly important to clinical care and the “outcomes and effectiveness” domain consistently scores the highest in assessment of capacity to sustain PEWS (Agulnik, Malone, et al., 2021). Other interventions with different characteristics may demonstrate sustainment is influenced by different capacity domains. Additionally, in alignment with our theoretical framework of sustainability, we expect interaction between the intervention and clinical capacity for sustainability to change over time. Future research should longitudinally explore the relationship between intervention characteristics and clinical capacity to help elucidate this interaction and guide strategies to optimize sustainability long-term.

As others have noted, very few empirically informed strategies to promote intervention sustainment are available (Shelton et al., 2018). Our process of evaluating clinical capacity determinants for sustainability and exploring their association with PEWS sustainment provides a model for helping identify modifiable factors that can be the target for development of novel sustainability strategies. For PEWS specifically, this may be strategies to identify dedicated resources for PEWS during the planning and implementation phases, and multidisciplinary stakeholder involvement to ensure PEWS is fully integrated into the clinical workflow as part of the pilot and implementation phase. More broadly, there are established compilations of strategies to help address important determinants. For instance, a recent revision of ERIC strategies, a compilation of implementation strategies initially intended to promote the adoption and initial implementation of evidence-based interventions, expanded implementation strategies to intervention sustainment. However, these proposed strategies have not been associated with specific sustainability determinants or tested empirically. We recommend conducting assessments such as the one described in this study to identify determinants impacting intervention sustainment and inform strategy selection to optimize sustainability.

### Strengths and Limitations

This analysis leveraged a cross-sectional research design which does not allow for determination of whether clinical capacity predicts PEWS sustainment. In addition, several other significant variables, e.g., length of time working at the hospital, may be better predictors of PEWS sustainment and explain why or why not PEWS may be sustained. The size of the study, however, with nearly 800 participants across 37 diverse centers in 13 countries all sustaining one intervention for multiple years, makes this study adequately powered to identify significant multi-level factors impacting PEWS sustainment across centers. Our high response rate added to the validity of these findings. Similarly, diversity of centers (with variable hospital structure, resource level, and size) allowed for examination of institutional structural factors impacting PEWS sustainment. However, we also recognize that hospitals from Mexico were heavily represented in our sample. This is in part due to the structure of the health system, which is relatively decentralized, and many hospitals provide cancer treatment compared to other countries in the sample. We encourage future work to use prospective, longitudinal designs to understand the dynamic interaction between intervention characteristics, adaptation, and sustainment over time and the contextual factors at multiple levels that may influence these relationships.

## Conclusion

We evaluated clinical capacity determinants impacting sustainment of an evidence-based practice, PEWS, across a diverse cohort of resource-variable pediatric oncology centers in Latin America. Our study demonstrated a strong relationship between capacity and intervention sustainment and identified multiple potential capacity factors integral to PEWS sustainment. PEWS is a highly sustainable evidence-based intervention that should be scaled up to reduce global disparities in childhood cancer survival.

## Supplementary Material

Supplementary Material

## Figures and Tables

**Table 1:** Participant and hospital characteristics

Participant characteristics	*n*	%	Hospital characteristics		
**Gender**			**World Bank Income Group (*N*, %)**		
Male	141	18	Low middle income	5	13.5
Female	656	82	Upper middle income	31	83.8
Other	0	0	High income	1	2.7
**Profession**			**Hospital type (*N*, %)**		
Nurse	455	57	General or women and children’s	16	43.2
Doctor	320	40	Pediatric multidisciplinary	14	37.8
Other	22	3	Oncology (adult and pediatric)	5	13.5
**Main Area of Work**			Pediatric oncology	2	5.4
Floor	692	87	**Funding type (*N*, %)**		
Intensive Care	70	9	Public	27	73.0
Other	35	4	Private	5	13.5
**PEWS Implementation Role**			Mixed (public and private)	5	13.5
PEWS Implementation Leader	161	20	**Teaching hospital (*N*, %)**		
Clinical Staff	574	72	Yes	35	94.6
Other	62	8	No	2	5.4
**Length of Work at Hospital**			**Annual new diagnoses (*M*, range)**	89	5–800
5 years or less	317	40	Nurse-to-patient ratio (*M*, range)		
6–10 years	215	27	**(1 nurse for every × patients on ward)**	6	3–10
More than 10 years	265	33	**Time since PEWS implementation completion (*M*, range)**	28	4–88
Total	797	100	Total (*N*, %)	37	100

**Table 2:** Intervention-level characteristics

	*n*	%
**Strength of evidence supporting PEWS**		
Not strong or unknown	82	10
Strong or very strong	715	90
**Importance of PEWS to providing quality care**		
Not important or neutral	14	2
Important or very important	783	98
**Difficulty of PEWS implementation**		
Difficult or somewhat difficult	279	35
Neither easy nor difficult	195	24
Easy or somewhat easy	287	36
Don’t know/NA	36	5
Total	797	100

**Table 3:** PEWS sustainment modeled with individual, hospital, intervention, and capacity variables

	Overall capacity		Individual capacity domains	
	Coef. (95% CI)	*P*	Coef. (95% CI)	*P*

Intercept	− 6.06 (− 9.52 to 2.60)	0.01	− 6.23 (− 9.69 to 2.77)	0.01

	Odds Ratio (95% CI)	*P*	Odds Ratio (95% CI)	*P*
Gender (ref: Male)		0.23		0.09
Female	1.45 (0.79 to 2.66)	0.23	1.72 (0.92 to 3.20)	0.09
Profession (ref: Nurse)		< .01		< .01
Doctor	0.24 (0.13 to 0.44)	< .01	0.23 (0.12 to 0.44)	< .01
Other	3.45 (0.28 to 42.30)	0.33	3.72 (0.3 to 46.42)	0.31
Main area of work (ref: Floor)		0.09		0.10
ICU	0.92 (0.41 to 2.09)	0.85	0.96 (0.42 to 2.24)	0.93
Other	0.23 (0.06 to 0.85)	0.03	0.23 (0.06 to 0.88)	0.03
Role on EVAT implementation team (ref: Clinical Staff)	0.08		0.13	
EVAT leader	2.14 (1.03 to 4.44)	0.04	2.01 (0.95 to 4.28)	0.07
Admin, data manager, or other	2.26 (0.66 to 7.74)	0.20	2.18 (0.65 to 7.32)	0.21
Length of work at hospital (ref: > 10 years)	0.03		0.04	
< = 5 years	2.53 (1.32 to 4.82)	0.05	2.34 (1.22 to 4.50)	0.01
6–10 years	2.77 (1.4 to 5.47)	0.04	2.93 (1.45 to 5.91)	0.03
World Bank income group (ref: HIC)	0.53		0.42	
LMIC	3.69 (0.31 to 44.65)	0.30	4.19 (0.37 to 47.73)	0.25
UMIC	2.30 (0.24 to 22.31)	0.47	2.39 (0.26 to 21.84)	0.44
Hospital type (ref: General or woman and children’s hospital)	0.20		0.16	
Oncology (adult and pediatric)	1.16 (0.29 to 4.61)	0.83	0.96 (0.25 to 3.77)	0.96
Pediatric oncology	1.27 (0.08 to 20.57)	0.87	1.12 (0.08 to 16.10)	0.93
Pediatric multidisciplinary	0.38 (0.15 to 0.97)	0.04	0.35 (0.14 to 0.89)	0.03
Funding type (ref: Private)		0.12		0.08
Mix (public/private)	5.86 (1 to 34.21)	0.05	6.51 (1.18 to 35.8)	0.03
Public	4.19 (0.89 to 19.69)	0.07	4.51 (1.02 to 19.92)	0.05
Teaching hospital (ref: No)		0.83		0.64
Yes	1.18 (0.27 to 5.19)	0.83	1.40 (0.34 to 5.72)	0.64
Annual New diagnoses	0.84 (0.65 to 1.09)	0.19	0.84 (0.65 to 1.08)	0.17
Nurse-to-Patient Ratio	0.93 (0.76 to 1.15)	0.51	0.96 (0.78 to 1.18)	0.68
Time Sustaining PEWS	1.02 (0.98 to 1.05)	0.37	1.02 (0.98 to 1.05)	0.35
PEWS intervention—strength of the scientific evidence (ref: Not strong)	0.11		0.14	
Strong	0.53 (0.24 to 1.15)	0.11	0.55 (0.25 to 1.22)	0.14
PEWS intervention—important to providing quality care (ref: Not important)	0.02		0.02	
Important	6.3 (1.43 to 27.77)	0.02	6.29 (1.36 to 29.10)	0.02
PEWS intervention—difficulty of implementation (ref: Neutral)	0.60		0.63	
Difficult	0.86 (0.45 to 1.66)	0.66	0.96 (0.49 to 1.90)	0.92
Easy	0.65 (0.34 to 1.26)	0.20	0.68 (0.35 to 1.34)	0.26
Unknown	0.66 (0.20 to 2.17)	0.49	0.77 (0.22 to 2.61)	0.67
Overall capacity score	3.27 (2.12 to 5.06)	< .01		
Individual capacity domains				
Engaged staff and leadership			0.95 (0.51 to 1.76)	0.87
Engaged stakeholders			0.63 (0.35 to 1.14)	0.13
Organizational Readiness			2.02 (1.21 to 3.39)	< 0.01
Workflow integration			2.70 (1.42 to 5.16)	< 0.01
Implementation and Training			1.49 (0.93 to 2.39)	0.10
Monitoring and Evaluation			0.77 (0.47 to 1.25)	0.29
Outcomes and Effectiveness			0.87 (0.52 to 1.45)	0.60
AIC	554.53		548.62	
