# Supplementary material for: Connecting Clinical Capacity and Intervention Sustainability in Resource-Variable Pediatric Oncology Centers in Latin America
Source: Glob Implement Res Appl. Author manuscript; Available in PMC 2024 Apr 2. (PMC10987010; doi:10.1007/s43477-023-00106-2)
Supplement: Supplementary Material [file NIHMS1969203-supplement-Supplementary_Material.docx]

|  | **(1) Null Model** | **(2) Individual-level variables** | | **(3) Hospital-level variables** | | **(4) Intervention-level variables** | | **(5) Overall capacity variable** | | **(6) Individual capacity domain variables** | |
| --- | --- | --- | --- | --- | --- | --- | --- | --- | --- | --- | --- |
|  | **Coef.**  **(95% CI)** | **Coef.**  **(95% CI)** | ***P*** | **Coef.**  **(95% CI)** | ***P*** | **Coef.**  **(95% CI)** | ***P*** | **Coef.**  **(95% CI)** | ***P*** | **Coef.**  **(95% CI)** | ***P*** |
| Intercept | 2.15  (1.73-2.57) | 1.71  (0.95-2.47) | <.0001 | -0.62  (-3.43-2.20) | 0.66 | -2.70  (-5.83-0.43) | 0.09 | -6.06  (-9.52--2.60) | 0.001 | -6.23  (-9.69--2.77) | 0.001 |
|  |  | **Odds Ratio**  **(95% CI)** | ***P*** | **Odds Ratio**  **(95% CI)** | ***P*** | **Odds Ratio**  **(95% CI)** | ***P*** | **Odds Ratio**  **(95% CI)** | ***P*** | **Odds Ratio**  **(95% CI)** | ***P*** |
| *Gender (ref: Male)* |  |  | 0.43 |  | 0.43 |  | 0.53 |  | 0.23 |  | 0.09 |
| Female |  | 1.26  (0.71-2.24) | 0.43 | 1.26  (0.71-2.25) | 0.43 | 1.21  (0.67-2.17) | 0.53 | 1.45  (0.79-2.66) | 0.23 | 1.72  (0.92-3.20) | 0.09 |
| *Profession (ref: Nurse)* |  |  | 0.0001 |  | 0.0004 |  | 0.0002 |  | <.0001 |  | <.0001 |
| Doctor |  | 0.32  (0.18-0.55) | <.0001 | 0.35  (0.20-0.61) | 0.0002 | 0.33  (0.19-0.58) | 0.0001 | 0.24  (0.13-0.44) | <.0001 | 0.23  (0.12-0.44) | <.0001 |
| Other |  | 3.83  (0.35-42.36) | 0.27 | 3.99  (0.36-44.33) | 0.26 | 3.85  (0.35-42.59) | 0.27 | 3.45  (0.28-42.30) | 0.33 | 3.72  (0.3-46.42) | 0.31 |
| *Main area of work (ref: Floor)* |  |  | 0.13 |  | 0.16 |  | 0.17 |  | 0.09 |  | 0.10 |
| ICU |  | 0.76  (0.36-1.63) | 0.49 | 0.74  (0.34-1.59) | 0.44 | 0.80  (0.37-1.75) | 0.57 | 0.92  (0.41-2.09) | 0.85 | 0.96  (0.42-2.24) | 0.93 |
| Other |  | 0.28  (0.08-0.99) | 0.05 | 0.31  (0.09-1.08) | 0.07 | 0.31  (0.09-1.08) | 0.07 | 0.23  (0.06-0.85) | 0.03 | 0.23  (0.06-0.88) | 0.03 |
| *Role on EVAT implementation team (ref: Clinical Staff)* |  |  | 0.02 |  | 0.02 |  | 0.03 |  | 0.08 |  | 0.13 |
| EVAT leader |  | 2.64  (1.33-5.25) | 0.006 | 2.58  (1.3-5.13) | 0.007 | 2.47  (1.22-4.97) | 0.01 | 2.14  (1.03-4.44) | 0.04 | 2.01  (0.95-4.28) | 0.07 |
| Admin, data manager, or other |  | 2.14  (0.68-6.77) | 0.20 | 2.18  (0.69-6.90) | 0.18 | 2.03  (0.63-6.48) | 0.23 | 2.26  (0.66-7.74) | 0.20 | 2.18  (0.65-7.32) | 0.21 |
| *Length of work at hospital (ref: >10 years)* |  |  | 0.0004 |  | 0.001 |  | 0.002 |  | 0.003 |  | 0.004 |
| <=5 years |  | 2.82  (1.57-5.08) | 0.0006 | 2.61  (1.43-4.78) | 0.002 | 2.66  (1.43-4.94) | 0.002 | 2.53  (1.32-4.82) | 0.005 | 2.34  (1.22-4.50) | 0.01 |
| 6-10 years |  | 2.96  (1.55-5.67) | 0.001 | 2.81  (1.45-5.43) | 0.002 | 2.82  (1.45-5.46) | 0.002 | 2.77  (1.4-5.47) | 0.004 | 2.93  (1.45-5.91) | 0.003 |
| *World Bank income group (ref: HIC)* |  |  |  |  | 0.43 |  | 0.43 |  | 0.53 |  | 0.42 |
| LMIC |  |  |  | 4.83  (0.4-58.63) | 0.22 | 4.89  (0.41-57.67) | 0.21 | 3.69  (0.31-44.65) | 0.30 | 4.19  (0.37-47.73) | 0.25 |
| UMIC |  |  |  | 2.97  (0.3-29.12) | 0.35 | 3.17  (0.33-30.09) | 0.31 | 2.30  (0.24-22.31) | 0.47 | 2.39  (0.26-21.84) | 0.44 |
| *Hospital type (ref: General or woman & children’s hospital)* |  |  |  |  | 0.14 |  | 0.11 |  | 0.20 |  | 0.16 |
| Oncology (adult & pediatric) |  |  |  | 1.55  (0.4-6.02) | 0.53 | 1.43  (0.37-5.51) | 0.60 | 1.16  (0.29-4.61) | 0.83 | 0.96  (0.25-3.77) | 0.96 |
| Pediatric oncology |  |  |  | 1.05  (0.07-16.88) | 0.97 | 1.04  (0.07-16.12) | 0.98 | 1.27  (0.08-20.57) | 0.87 | 1.12  (0.08-16.10) | 0.93 |
| Pediatric multidisciplinary |  |  |  | 0.38  (0.15-0.97) | 0.04 | 0.35  (0.14-0.89) | 0.03 | 0.38  (0.15-0.97) | 0.04 | 0.35  (0.14-0.89) | 0.03 |
| *Funding type (ref: Private)* |  |  |  |  | 0.26 |  | 0.15 |  | 0.12 |  | 0.08 |
| Mix (public/private) |  |  |  | 3.61  (0.66-19.91) | 0.14 | 5.17  (0.91-29.24) | 0.06 | 5.86  (1-34.21) | 0.05 | 6.51  (1.18-35.8) | 0.03 |
| Public |  |  |  | 3.40  (0.74-15.65) | 0.12 | 3.86  (0.84-17.63) | 0.08 | 4.19  (0.89-19.69) | 0.07 | 4.51  (1.02-19.92) | 0.05 |
| *Teaching hospital (ref: No)* |  |  |  |  | 0.56 |  | 0.57 |  | 0.83 |  | 0.64 |
| Yes |  |  |  | 1.55  (0.35-6.79) | 0.56 | 1.52  (0.36-6.47) | 0.57 | 1.18  (0.27-5.19) | 0.83 | 1.40  (0.34-5.72) | 0.64 |
| Annual New diagnoses |  |  |  | 0.76  (0.59-0.98) | 0.03 | 0.77  (0.59-0.99) | 0.04 | 0.84  (0.65-1.09) | 0.19 | 0.84  (0.65-1.08) | 0.17 |
| Nurse-to Patient Ratio |  |  |  | 0.94  (0.76-1.15) | 0.54 | 0.93  (0.76-1.14) | 0.50 | 0.93  (0.76-1.15) | 0.51 | 0.96  (0.78-1.18) | 0.68 |
| Time Sustaining PEWS |  |  |  | 1.02  (0.99-1.05) | 0.23 | 1.02  (0.99-1.05) | 0.22 | 1.02  (0.98-1.05) | 0.37 | 1.02  (0.98-1.05) | 0.35 |
| *PEWS intervention - strength of the scientific evidence (ref: Not strong)* |  |  |  |  |  |  | 0.63 |  | 0.11 |  | 0.14 |
| Strong |  |  |  |  |  | 0.83  (0.4-1.74) | 0.63 | 0.53  (0.24-1.15) | 0.11 | 0.55  (0.25-1.22) | 0.14 |
| *PEWS intervention – important to providing quality care (ref: Not important)* |  |  |  |  |  |  | 0.001 |  | 0.02 |  | 0.02 |
| Important |  |  |  |  |  | 11.14  (2.64-46.92) | 0.001 | 6.3  (1.43-27.77) | 0.02 | 6.29  (1.36-29.10) | 0.02 |
| *PEWS intervention – difficulty of implementation (ref: Neutral)* |  |  |  |  |  |  | 0.86 |  | 0.60 |  | 0.63 |
| Difficult |  |  |  |  |  | 0.81  (0.43-1.53) | 0.51 | 0.86  (0.45-1.66) | 0.66 | 0.96  (0.49-1.90) | 0.92 |
| Easy |  |  |  |  |  | 0.81  (0.43-1.52) | 0.51 | 0.65  (0.34-1.26) | 0.20 | 0.68  (0.35-1.34) | 0.26 |
| Unknown |  |  |  |  |  | 0.67  (0.21-2.12) | 0.49 | 0.66  (0.20-2.17) | 0.49 | 0.77  (0.22-2.61) | 0.67 |
| Overall CSAT score |  |  |  |  |  |  |  | 3.27  (2.12-5.06) | <.0001 |  |  |
| Engaged staff and leadership |  |  |  |  |  |  |  |  |  | 0.95  (0.51-1.76) | 0.87 |
| Engaged stakeholders |  |  |  |  |  |  |  |  |  | 0.63  (0.35-1.14) | 0.13 |
| Organizational Readiness |  |  |  |  |  |  |  |  |  | 2.02  (1.21-3.39) | 0.008 |
| Workflow integration |  |  |  |  |  |  |  |  |  | 2.70  (1.42-5.16) | 0.003 |
| Implementation and Training |  |  |  |  |  |  |  |  |  | 1.49  (0.93-2.39) | 0.10 |
| Monitoring & Evaluation |  |  |  |  |  |  |  |  |  | 0.77  (0.47-1.25) | 0.29 |
| Outcomes and Effectiveness |  |  |  |  |  |  |  |  |  | 0.87  (0.52-1.45) | 0.60 |
| **AIC** | **602.51** | **573.07** | | **584.95** | | **582.69** | | **554.53** | | **548.62** | |
